# Protein-Protein Fusion Catalyzed by Sortase A

**DOI:** 10.1371/journal.pone.0018342

**Published:** 2011-04-06

**Authors:** David A. Levary, Ranganath Parthasarathy, Eric T. Boder, Margaret E. Ackerman

**Affiliations:** 1 Thayer School of Engineering, Dartmouth College, Hanover, New Hampshire, United States of America; 2 Department of Physics, Harvard University, Cambridge, Massachusetts, United States of America; 3 Departments of Chemical and Biomolecular Engineering, University of Tennessee, Knoxville, Tennessee, United States of America; City of Hope National Medical Center and Beckman Research Institute, United States of America

## Abstract

Chimeric proteins boast widespread use in areas ranging from cell biology to drug delivery. Post-translational protein fusion using the bacterial transpeptidase sortase A provides an attractive alternative when traditional gene fusion fails. We describe use of this enzyme for *in vitro* protein ligation and report the successful fusion of 10 pairs of protein domains with preserved functionality — demonstrating the robust and facile nature of this reaction.

## Introduction

The ability to incorporate disparate modular domains within multi-functional chimeric proteins has revolutionized both the basic and applied biological sciences. While genetic fusion remains the gold-standard for chimera production, a given pair of protein domains may fail to express in tandem either in reasonable yield, or with proper folding and function—thereby limiting the diversity of domains that can be combined. Fusion protein expression can be particularly difficult when working with recombinant proteins originating from different hosts.

Post-translational protein fusion would allow native expression of the individual fusion partners—permitting expression of each domain in the optimum host, as well as allowing modular pairing and assembly of component domains after expression—effectively circumventing the diversity-limiting cloning steps of tandem genetic fusion. A number of systems for protein-protein fusion have been explored, including native chemical ligation [1], intein and enzyme based strategies [2], [3], and residue specific chemistries relying on cysteines or unnatural amino acids [4]. Unfortunately, these solutions tend to be technically challenging, residue rather than site specific, or necessitate the inclusion of large additional protein domains to mediate fusion.

Staphylococcal Sortase A is a bacterial transpeptidase that covalently attaches proteins to the bacterial cell wall by cleaving between threonine and glycine at an LPXTG recognition motif to generate an acyl-enzyme intermediate which then reacts with an N-terminal glycine, regenerating a native amide bond [5], [6]. This chemistry has been increasingly exploited to site-specifically link proteins displaying the C-terminal LPETGX_n_ motif to a range of substituents possessing a glycine or aminomethylene motif [7], including fluorophores [8], [9], photoaffinity probes [8], peptide nucleic acids [10], sugars [11], polymers [12], solid supports [12], [13], [14], lipids [15], [16], microsperes [16], [17], and enzymes utilized in enzyme-linked immunosorbent assays (ELISA) [18]. Conjugation of green fluorescent protein (GFP) to cell surfaces [9] and to make GFP multimers has been demonstrated under reducing conditions [7], [12]. Additional use as a means to circularize proteins [19] and in domain-specific NMR [20], demonstrates the wide range of sortase-catalyzed reactions. These reports, as well as recent reviews [21], [22], [23], reveal the broad applicability of sortase A catalyzed addition reactions, and motivated further study of its use and optimization in producing complex fusion proteins.

## Materials and Methods

### Protein Expression and Purification

Sortase-His6 enzyme was expressed essentially as described previously [12]. Briefly, BL21 E. coli (Invitrogen, Carlsbad, CA) transformed with pHTT27 [24] (a gift of Dr. Olaf Schneewind, University of Chicago) were induced with 1 mM isopropyl-ß-D-1-thiogalactopyranoside (IPTG) for 4 hours after reaching an OD_600_ of 0.4. Cytosolic proteins were extracted with B-per reagent (Pierce, Rockford, IL) from pelleted cells that had been frozen overnight. Sortase was purified from lysate using Talon resin (Clontech, Mountain View, CA), and buffer exchanged into 50 mM Tris, 150 mM NaCl, pH 8 using a 10,000 MW cutoff spin filter (Pierce, Rockford, IL).

A33 antigen extracellular domain bearing a His_6_ tag (A33-LPETG-His_6_), and all IgG constructs were produced in HEK293 cells (Invitrogen, Carlsbad, CA) after PEI transfection with gWIZ plasmids as described [25], [26] (Genlantis, San Diego, CA) according to the manufacturer's instructions. Secreted IgG was purified by Protein A chromatography (Pierce, Rockford, IL), and His_6_ tagged protein by affinity chromatography using Talon resin (Clontech, Mountainview, CA). GGG-GFP was produced in BL21 E. coli as described [12]. GGG-Gelonin, GGG-Fab, and GGG-albumin were kind gifts from Christopher Pirie, Mike Schmidt, and Kelly Davis Orcutt and produced in YVH10 yeast utilizing pRS shuttle vectors as described [27], [28]. All protein sequences are described in Supporting Information S1.

### Fusion Pair Tests

A33 antigen-LPETG-His_6_ and IgG-LC-LPETGGS at micromolar concentrations were reacted with each of the following: GGG-GFP, GGG-Fab, GGG-IgG, GGG-gelonin, and GGG-albumin (also at micromolar concentrations). Reactions were allowed to proceed for 2 hours at 37°C after addition of 10× reaction buffer (50 mM Tris, 150 mM NaCl, pH 8, 60 mM CaCl_2_) and 100 nM sortase enzyme.

The resulting samples were analyzed by running at 200 V on 12% bis-tris or Tris-acetate gels (Invitrogen, Carlsbad, CA) according the manufacturer's protocol after the addition of 4× sample dye (Invitrogen, Carlsbad, CA), as appropriate, after being boiled for 10 min. Proteins were visualized by staining with Simply Blue Safestain (Invitrogen, Carlsbad, CA).

For Western blots, gels were transferred onto nitrocellulose at 50 V in Invitrogen transfer buffer and blocked in 5% milk in phosphate buffered saline with 0.1% Tween-20. An anti-human light chain-HRP conjugate (AbD Serotec, Raleigh, NC) was used to detect the light chain in the IgG-LC-LPETG fusions, and a murine antibody to human A33 antigen (gift of Gerd Ritter, Ludwig Institute, New York) and goat anti-mouse-HRP (Sigma, St. Louis, MO) were used to detect A33 antigen before and after sortase reaction. Bands were detected using ECL reagents (Pierce, Rockford, IL), and where appropriate, quantified using ImageJ (NIH, Bethesda, MD).

### Functional Testing

For functional tests of the A33 binding IgG-LC-LPETGGS fused to GFP, 1 µl of Dynabeads® Biotin Binder magnetic beads (Dynal, Invitrogen, Carlsbad, CA) was washed twice in phosphate buffered saline, 0.1% bovine serum albumin (PBSA), and incubated with 250 µl of 850 nM biotinylated A33 antigen (biotinylation reagent, Pierce, Rockford, IL) in PBSA at 4°C overnight on a rotator. After beads were washed twice in PBSA to remove free antigen, 10 µl of IgG-GFP sortase reaction mixture was added to the antigen coated beads. The reaction mix and beads were incubated overnight at 4°C on a rotator, then washed twice before being analyzed for GFP signal by flow cytometry on a Coulter EpicsXL (Beckman Coulter, Fullerton, CA). Bare beads and a mock reaction mix excluding sortase were run as negative controls, and the binding of IgG-GFP was specifically competed using an excess of soluble antibody. Because both the IgG and GFP are conformationally sensitive, these tests demonstrate function and proper folding of both domains.

For functional tests of CEA (carcinoembryonic antigen) binding IgG fused to A33 antigen, a similar protocol was followed utilizing biotinylated CEA (antibody, Fitzgerald Industries, Concord, MA; biotinylation, Pierce, Rockford, IL) to coat beads, and an Alexa-488 labeled anti-A33 IgG (antibody gift of Gerd Ritter, Ludwig Institute, New York; Alexa labeling, Molecular Probes, Invitrogen, Carlsbad, CA) to detect A33 antigen. Binding of fusion product was specifically competed using as excess of soluble CEA-IgG.

Equilibrium titrations of these IgG fusion proteins were conducted as described previously [26]. Briefly, fixed LS174T cells, expressing both A33 and CEA antigens, were incubated in excess fusion protein at varying concentrations and detected by flow cytometry using anti-human IgG-PE. Binding affinities were determined and compared to those of non-fused IgGs.

### Reaction Optimization

The ligation reaction was optimized using A33-LPETG-His_6_ and GGG-GFP. Briefly, 20 µl test reactions were conducted under a variety of conditions. Each reaction consisted of LPETGX_n_ and GGG reactants, sortase A, in storage buffer (50 mM Tris, 150 mM NaCl, pH 8) and 10× reaction buffer (60 mM CaCl_2_ in 1× storage buffer). Reactant concentrations ranged from 5 to 50 uM, while enzyme concentrations were varied from 50 nM to 250 uM. The gradient function of a thermocycler was used to vary reaction temperature, and reaction time was varied by removing aliquots of a large reaction mix at set intervals, chilling to 4°C, and adding EDTA to a final concentration of 10 mM to stop the reaction. The resulting samples were analyzed by electrophoresis at 200 V on 12% bis-tris or Tris-acetate gels (Invitrogen, Carlsbad, CA) according the manufacturer's protocol after the addition of 4× sample dye and 10× reducing agent (Invitrogen, Carlsbad, CA), as appropriate, before being boiled for 10 min. The fusion product, approximate MW 70 kDa, was visualized after staining with Simply Blue Safestain (Invitrogen, Carlsbad, CA).

## Results and Discussion

### Sortassembly Method

**Figure 1:**
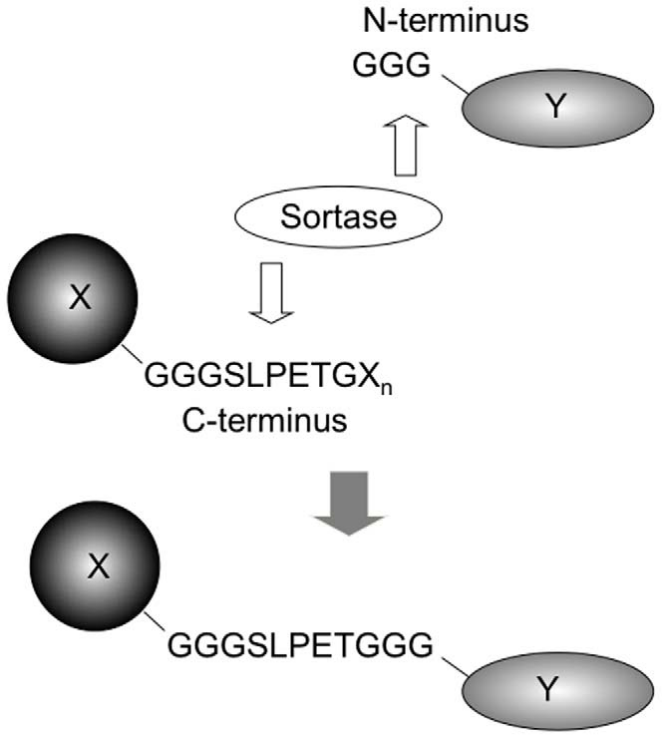
Schematic illustration of sortase A mediated reaction fusing two proteins. Sortase A fuses an LPXTG recognition motif to an N-terminal GGG motif, regenerating a native amide bond.

**Figure 2:**
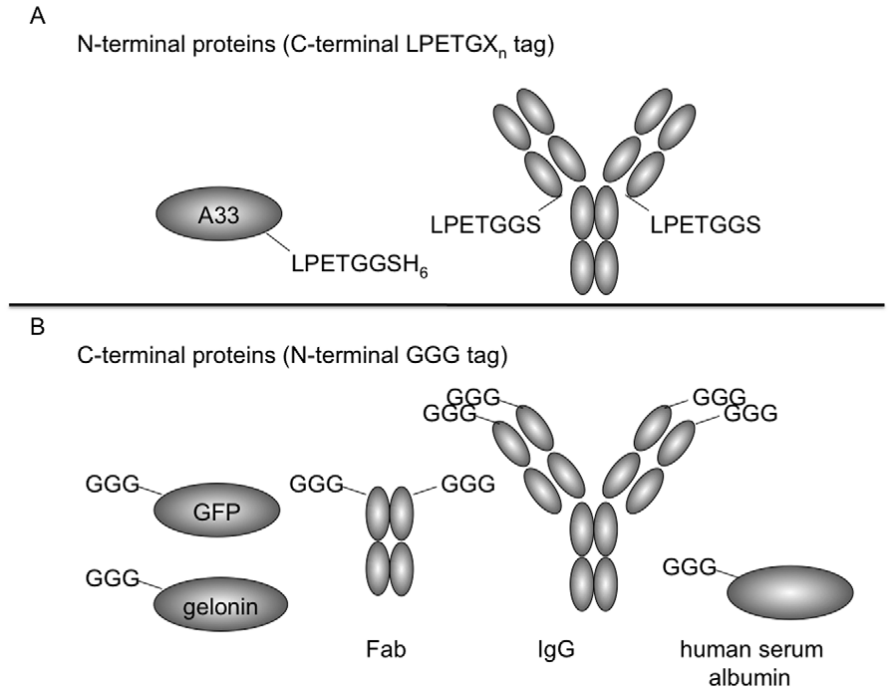
Structures of N and C-terminal fusion partners denoting site and sequence of sortase A recognition motifs of each domain.

We describe here tractable *in vitro* conditions for the site-specific assembly of diverse, multi-functional fusion proteins. Sortassembly requires only 2 short peptide tags: a C-terminal LPETGX_n_ tag on one partner, usually incorporated as LPETG-His_6_ to facilitate downstream separation, and a complementary N-terminal triglycine (GGG) motif on the other domain (Fig. 1). These domains are small and generally unobtrusive to protein production, yet allow site and stoichiometric control of the fusion reaction. Furthermore, a suite of proteins may be expressed with these tags allowing for modular pairing of diverse domains without additional molecular cloning effort. Figure 2 presents the structures of the protein domains used in this study.

### Testing the Diversity of Protein Domain Pairings

**Figure 3:**
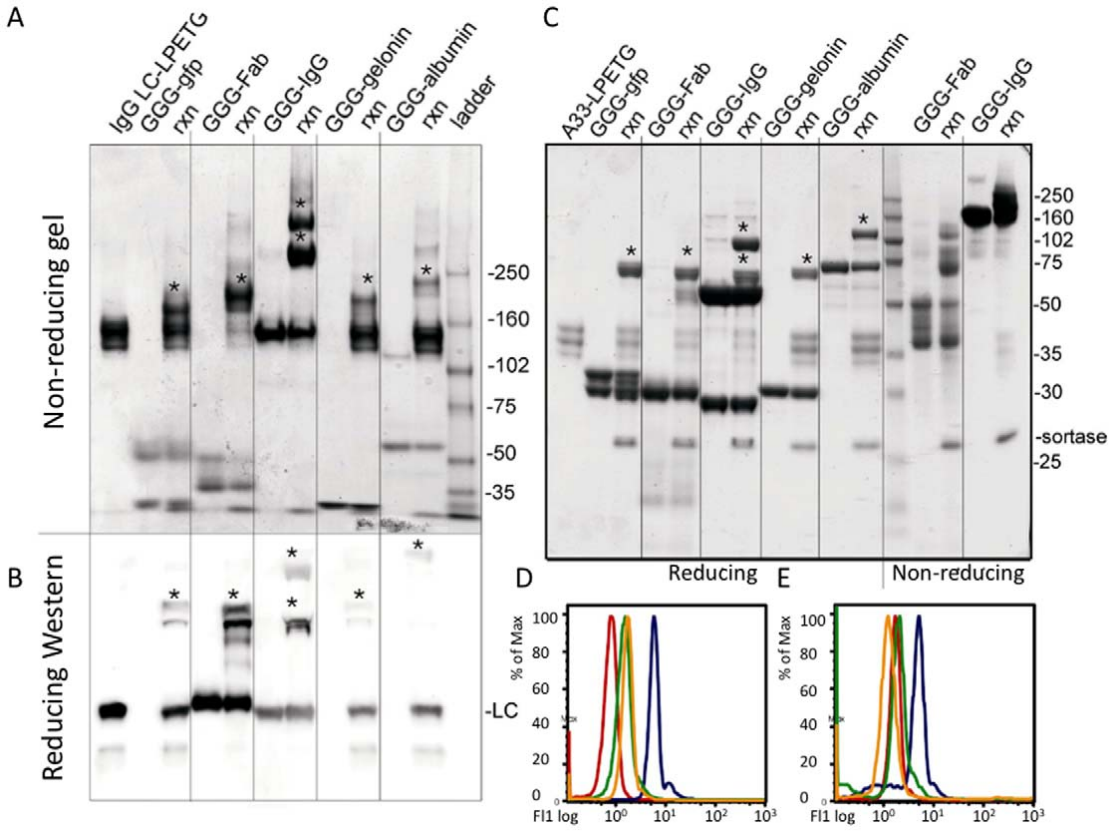
Sortassembled products and functional testing. Nonreduced gel (A) and reduced Western blot (B) demonstrating the shift in molecular weight of an IgG light chain-LPETGX_n_ following fusion with 5 different triglycine partners. Fusion protein products are marked with an asterisk. Light Chain (LC) was visualized. C) Gel demonstrating shift of A33 antigen-LPETGX_n_ following fusion in reactions (rxn) with 5 different triglycine partners. Fusion products are marked with an asterisk. D,E) Functional tests: fluorescence histograms of A33 IgG fused to GFP (D), and CEA IgG fused to A33 antigen (E). Blue trace represents reaction mix, remaining traces represent bare beads (red), reaction mix without sortase enzyme (green), and reaction mix with competitor IgG (yellow).

To assess the diversity of pairings that could be produced through sortase-catalyzed fusion reactions, we generated GGG- and LPETGX_n_ derivatives of a variety of representative modular domains, including enzymes (gelonin), antibodies (IgG, Fab), fluorescent protein (GFP), and cell surface and blood proteins (A33 antigen and human serum albumin) (Fig. 2), produced in a range of hosts, including E. coli, yeast, and mammalian cells. IgG fusions (Fig. 3A,B) and A33 fusions were produced with all 5 C-terminal fusion partners successfully. All reactions yielded fusion proteins of the appropriate molecular weight with yields ranging from approximately 30–85%. Particularly important given the growing interest in enzyme conjugated and bispecific antibody therapeutics was the successful ligation of whole antibodies without disruption of the associations between their heavy and light chains or constant domains, and the fusion of multiple triglycine reactants to substrates with multiple LPETG motifs (Fig. 3A). These results demonstrate the fusion of complex disulfide stabilized protein domains, and in conjunction with the numerous pairings tested, demonstrate that sortase A-mediated protein-protein fusion is a useful tool in the generation of fusion proteins when conventional genetic fusion fails.

### Functionality of Sortassembled Proteins

To demonstrate the preserved function of sortase-assembled fusion proteins, magnetic beads were coated with antigen and then incubated with reaction mixtures containing IgG-based fusion proteins: A33 IgG fused to GFP, and CEA IgG fused to A33 antigen. The protein fused to the IgG was then detected by flow cytometry either directly due to inherent fluorescence (detection of GFP), or by incubation with a conformation-specific antibody-fluorophore conjugate (detection of A33 antigen). In both cases, high signal intensity was observed, and signal decreased in the presence of specific competitor (Fig. 3D,E), indicating that the conformation and functionality of both fusion partners remained intact following sortassembly. Additionally, titrations of these sortassembled IgG fusions demonstrated the retention of affinity of the IgG domains (data not shown), providing further support as to the mild nature of the sortassembly reaction.

### Reaction Optimization

In order to increase the usefulness of this method, we sought to optimize reaction conditions in order to maximize product yield. The reaction is typically carried out in pH 8.2 buffered Tris containing 6 mM CaCl_2_ and 150 mM NaCl. We systematically varied and optimized reaction conditions using A33 antigen as a model LPETG-containing substrate and triglycine-GFP as its fusion partner. This optimization was done in reaction volumes as low as 20 µl before determination of ideal conditions to be used in large-scale reactions. Overall, the reaction was robust and fusion product yield was high under a broad range of conditions, permitting the stability and sensitivities of the proteins being ligated to determine reaction conditions rather than a narrow catalytic window. Briefly, ligation product was maximal under slightly alkaline conditions with high substrate concentrations in a reaction at 42°C for up to 4 hours with dilute sortase A.

**Figure 4:**
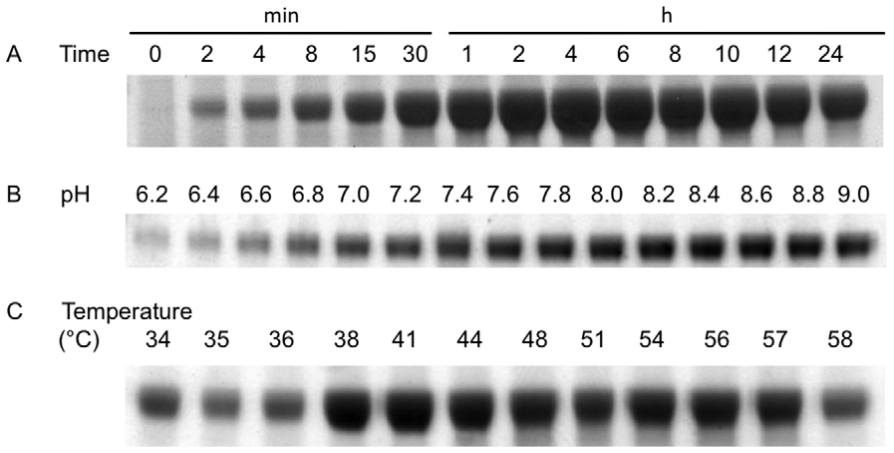
Optimizing reaction conditions. A) Fusion protein yield in a reaction time course demonstrating maximal yield between 1 and 4 hours, and reduced yield at prolonged reaction times. B) Product yield at varying pH demonstrating good yield at pH values between 7 and 9. C) Yield at different temperatures, demonstrating improved yield at temperatures slightly above 37°C.

Experimentally, the optimal reaction time is between 1 and 4 hours (Fig. 4A), after which EDTA may be added to quench the reaction. At long timepoints, a decrease in product is observed due to a competing hydrolysis reaction in which enzyme irreversibly hydrolyzes the LPETG motif found in both substrate and product. For proteins with pH sensitive conformations, the pH of the reaction may be varied between 7.0 and 9.0 while maintaining high yields (Fig. 4B). Perhaps surprisingly, as sortase A functions natively at 37°C, higher temperatures can produce a greater yield of fusion product (Fig. 4C), and are recommended as long as the proteins being fused are not thermolabile.

**Figure 5:**
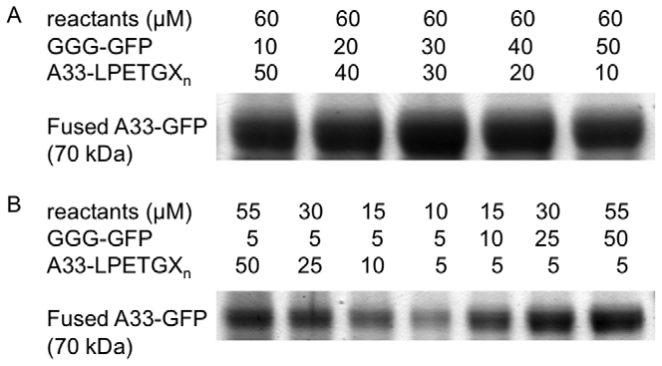
Optimizing concentrations of reactants. A) Fusion protein yield at differing ratios of reactants indicating that high concentrations of both reactants ought to be used. B) Gel of fusion product demonstrating that excess of either reactant pushes reaction toward completion.

High concentrations of reactants favor high yields (Fig. 5A) and the reaction may be pushed toward completion by adding an excess of either fusion partner (Fig. 5B). At a 1∶1 molar ratio of reactants, typical fusion protein yields range from 30–65% of the maximum theoretical yield, while with a 20-fold excess of one reactant, the reaction can proceed to approximately 85% yield as quantified by Western blot.

**Figure 6:**
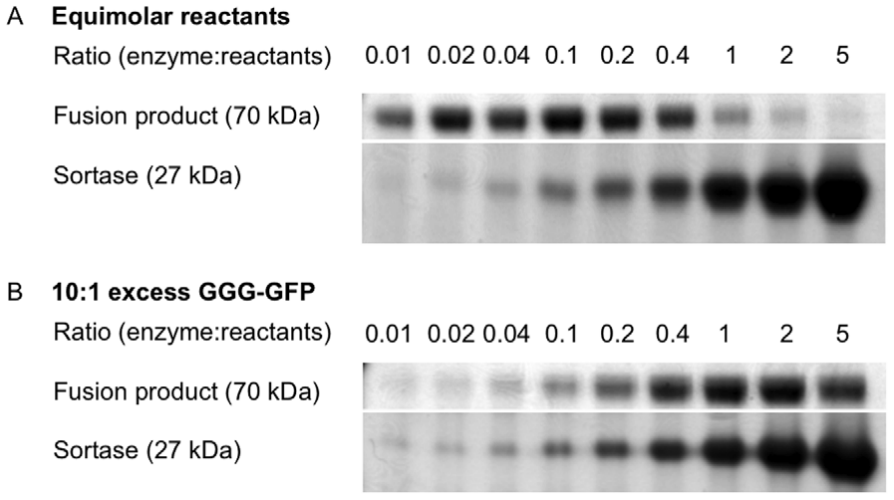
Optimizing enzyme concentrations. A) Fusion product yield in reactions with equimolar reactants, while varying the ratio of sortase∶total reactants. B) Fusion product yield with varying amounts of sortase enzyme in reactions with a 10-fold excess of triglycine reactant over LPETG reactant.

Care must be taken, however, in tailoring the concentration of sortase enzyme, as adding excess enzyme reduces the amount of fusion product either by inhibiting formation of the ternary complex, or by increasing the overall rate of an irreversible hydrolysis reaction of the LPETG motif (whether present in reactants or ligated product), which releases LPET as a terminal product. For equimolar fusion partners, a 0.1 molar equivalent of enzyme gave optimal fusion product yield (Fig. 6A)—although this fraction changes as the ratio of reactants is varied (Fig. 6B). Similarly, this hydrolysis reaction impacts the time allowed for the reaction to proceed. Because the LPETG motif is present in the desired fusion product, the enzyme will continue to react with fusion product, and over extended periods of time the irreversible hydrolysis reaction will outweigh any marginal increases in product yield as all substrate and product become hydrolyzed. Table 1 presents a summary of optimized reaction conditions.

**Table 1:** Optimized conditions for sortassembly.

Condition	Recommendation	Acceptable Range
Time	1–4 hours	30 min to 12 hours
Temperature	42°C	37–48°C
Enzyme	0.1–1 molar equivalent of total reactants	Depends on reactant ratio
Reactants	Micromolar concentration	nM and greater
pH	8.2	6.8–9.0

### Product Yield and Separation

**Figure 7:**
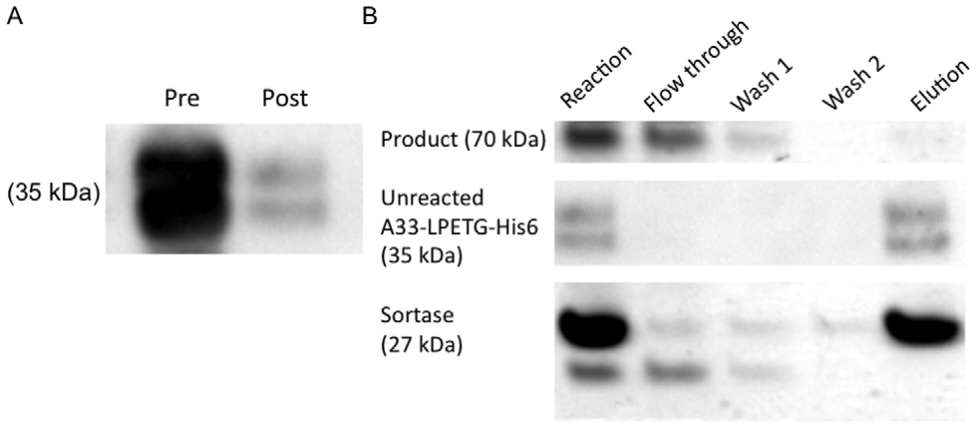
Product quantification and purification. A) Western blot showing amount of unreacted LPETGX_n_ substrate before (left) and after (right) reaction, demonstrating an 85% yield when bands are quantified. B) Example purification demonstrating separation of unreacted A33-LPETG-His_6_ and sortase-His_6_ enzyme from reaction mixture by nickel-based affinity chromatography.

In an optimized fusion reaction of A33 and GFP yields of 80% were attained, as determined by quantitative Western blotting (Fig. 7A). For other fusion pairs, in non-optimized reactions, yields ranged from 8–50%, while after optimization, yields ranged from 40–85%. An average 4-fold improvement in yield was observed, though in some reactions yield was improved as much as 7-fold. For separation of product from reactants and enzyme, a His_6_ tag can be incorporated both after the LPETG motif and on the sortase enzyme, allowing unreacted substrate and enzyme to be removed simply by passing over an affinity column (Fig. 7B). Product may then be separated from the tri-glycine reactant by methods such as size exclusion, ion exchange, or affinity chromatography. In optimized reactions we have been able to achieve 80% yields of fusion protein, with reagents and enzyme undetectable after downstream separations.

### Conclusion

By decoupling protein expression and fusion, and allowing native amide bonds to be formed through inclusion of short peptide tags with site and stoichiometric control, sortase A-catalyzed assembly promises to increase the diversity of fusion proteins and the ease with which they can be synthesized. The mild and robust nature of this reaction, combined with its scalability allows immediate practical application.

## Supporting Information

Supporting Information S1 Provides the sequences including N and C-terminal sites recognized by sortase A for all proteins fused in this report.
(DOCX)
