# Supplementary material for: Protein-Protein Fusion Catalyzed by Sortase A
Source: PLoS One. 2011 Apr 6;6(4):e18342. doi: 10.1371/journal.pone.0018342 (PMC3071835; doi:10.1371/journal.pone.0018342)
Supplement: Supporting Information S1 — Provides the sequences including N and C-terminal sites recognized by sortase A for all proteins fused in this report. (DOCX) [file pone.0018342.s001.docx]

**Supplementary Information**

*Sequences of LPETGXn and GGG-containing Proteins*

A33-LPETG-His6 –

ISVETPQDVLRASQGKSVTLPCTYHTSTSSREGLIQWDKLLLTHTERVVIWPFSNKNYIHGELYKNRVSISNNAEQSDASITIDQLTMADNGTYECSVSLMSDLEGNTKSRVRLLVLVPPSKPECGIEGETIIGNNIQLTCQSKEGSPTPQYSWKRYNILNQEQPLAQPASGQPVSLKNISTDTSGYYICTSSNEEGTQFCNITVAVRSPSMNGGGGSGGGGS**LPETG**GSGHHHHHH

Anti-A33 IgG LC LPETG –

SELQMTQSPSSLSASVGDRVTITCLASEFLFNGVSWYQQKPGKVPKFLIYGASNLESGVPSRFSGSGSGTDFTLTISSLQPEDVATYYCLGGYSGSSGLTFGGGTKVEIKRTVAAPSVFIFPPSDEQLKSGTASVVCLLNNFYPREAKVQWKVDNALQSGNSQESVTEQDSKDSTYSLSSTLTLSKADYEKHKVYACEVTHQGLSSPVTKSFNRGECGGGGSGGGGS**LPETG**GSG

Anti-A33 IgG HC –

EVQVMESGGGLVKPGGSLRLSCAASGIGFSHYGISWVRQAPGKGLEWVAYIYPNYGSVDYASSVNGRFTISLDNAQNSLYLQMNSLRAEDTAVYFCARDRGYYSGSRGTRLDLWGQGTLVTVSSASTKGPSVFPLAPSSKSTSGGTAALGCLVKDYFPEPVTVSWNSGALTSGVHTFPAVLQSSGLYSLSSVVTVPSSSLGTQTYICNVNHKPSNTKVDKKVEPKSCDKTHTCPPCPAPELLGGPSVFLFPPKPKDTLMISRTPEVTCVVVDVSHEDPEVKFNWYVDGVEVHNAKTKPREEQYNSTYRVVSVLTVLHQDWLNGKEYKCKVSNKALPAPIEKTISKAKGQPREPQVYTLPPSRDELTKNQVSLTCLVKGFYPSDIAVEWESNGQPENNYKTTPPVLDSDGSFFLYSKLTVDKSRWQQGNVFSCSVMHEALHNHYTQKSLSLSPGK

GGG-GFP –

**GGG**LNDIFEAQKIEWHEMVSKgeelftgvvpilveldgdvnghkfsvsgegegdatygkltlkficttgklpvpwptlvttltygvqcfsrypdhmkqhdffksampegyvqertiffkddgnyktraevkfegdtlvnrielkgidfkedgnilghkleynynshnvyimadkqkngikvnfkirhniedgsvqladhyqqntpigdgpvllpdnhylstqsalskdpnekrdhmvllefvtaagitlgmdelykLEHHHHHH

Anti-CEA GGG-IgG LC –

**GGGGG**SENVLTQSPSSMSVSVGDRVTIACSASSSVPYMHWLQQKPGKSPKLLIYLTSNLASGVPSRFSGSGSGTDYSLTISSVQPEDAATYYCQQRSSYPLTFGCGTKLEIKATVAAPSVFIFPPSDEQLKSGTASVVCLLNNFYPREAKVQWKVDNALQSGNSQESVTEQDSKDSTYSLSSTLTLSKADYEKHKVYACEVTHQGLSSPVTKSFNRGEC

Anti-CEA GGG-IgG HC –

**GGGGG**SQVKLEQSGAEVVKPGASVKLSCKASGFNIKDSYMHWLRQGPGQCLEWIGWIDPENGDTEYAPKFQGKATFTTDTSANTAYLGLSSLRPEDTAVYYCNEGTPTGPYYFDYWGQGTLVTVSSPSTKGPSVFPLAPSSKSTSGGTAALGCLVKDYFPEPVTVSWNSGALTSGVHTFPAVLQSSGLYSLSSVVTVPSSSLGTQTYICNVNHKPSNTKVDKKVEPKSCDKTHTCPPCPAPELLGGPSVFLFPPKPKDTLMISRTPEVTCVVVDVSHEDPEVKFNWYVDGVEVHNAKTKPREEQYNSTYRVVSVLTVLHQDWLNGKEYKCKVSNKALPAPIEKTISKAKGQPREPQVYTLPPSRDELTKNQVSLTCLVKGFYPSDIAVEWESNGQPENNYKTTPPVLDSDGSFFLYSKLTVDKSRWQQGNVFSCSVMHEALHNHYTQKSLSLSPGK

Anti-CEA GGG-Fab LC –

**GGGGG**SENVLTQSPSSMSVSVGDRVTIACSASSSVPYMHWLQQKPGKSPKLLIYLTSNLASGVPSRFSGSGSGTDYSLTISSVQPEDAATYYCQQRSSYPLTFGCGTKLEIKATVAAPSVFIFPPSDEQLKSGTASVVCLLNNFYPREAKVQWKVDNALQSGNSQESVTEQDSKDSTYSLSSTLTLSKADYEKHKVYACEVTHQGLSSPVTKSFNRGEGSHHHHHH

Anti-CEA GGG-Fab HC –

**GGGGG**SQVKLEQSGAEVVKPGASVKLSCKASGFNIKDSYMHWLRQGPGQCLEWIGWIDPENGDTEYAPKFQRKATFTTDTSANTAYLGLSSLRPEDTAVYYCNEGTPTGPYYFDYWGQGTLVTVSAGTKGPSVFPLAPSSKSTSGGTAALGCLVKDYFPEPVTVSWNSGALTSGVHTFPAVLQSSGLYSLSSVVTVPSSSLGTQTYICNVNHKPSNTKVDKKVEPKSNASGWSHPQFEK

GGG-gelonin –

**GGG**ISEFGSSRVDLQGLDTVSFSTKGATYITYVNFLNELRVKLKPEGNSHGIPLLRKKCDDPGKCFVLVALSNDNGQLAEIAIDVTSVYVVGYQVRNRSYFFKDAPDAAYEGLFKNTIKTRLHFGGSYPSLEGEKAYRETTDLGIEPLRIGIKKLDENAIDNYKPTEIASSLLVVIQMVSEAARFTFIENQIRNNFQQRIRPANNTISLENKWGKLSFQIRTSGANGMFSEAVELERANGKKYYVTAVDQVKPKIALLKFVDK DPKAS

GGG-albumin –

**GGGGG**SDAHKSEVAHRFKDLGEENFKALVLIAFAQYLQQCPFEDHVKLVNEVTEFAKTCVADESAENCDKSLHTLFGDKLCTVATLRETYGEMADCCAKQEPERNECFLQHKDDNPNLPRLVRPEVDVMCTAFHDNEETFLKKYLYEIARRHPYFYAPELLFFAKRYKAAFTECCQAADKAACLLPKLDELRDEGKASSAKQRLKCASLQKFGERAFKAWAVARLSQRFPKAEFAEVSKLVTDLTKVHTECCHGDLLECADDRADLAKYICENQDSISSKLKECCEKPLLEKSHCIAEVENDEMPADLPSLAADFVESKDVCKNYAEAKDVFLGMFLYEYARRHPDYSVVLLLRLAKTYETTLEKCCAAADPHECYAKVFDEFKPLVEEPQNLIKQNCELFEQLGEYKFQNALLVRYTKKVPQVSTPTLVEVSRNLGKVGSKCCKHPEAKRMPCAEDYLSVVLNQLCVLHEKTPVSDRVTKCCTESLVNRRPCFSALEVDETYVPKEFNAETFTFHADICTLSEKERQIKKQTALVELVKHKPKATKEQLKAVMDDFAAFVEKCCKADDKETCFAEEGKKLVAASQAALGL
